# Impact of PD‐1 Inhibitor Timing Relative to Locoregional Therapy on Outcomes in uHCC Treated With Lenvatinib‐Based Triple Therapy

**DOI:** 10.1111/liv.70847

**Published:** 2026-08-27

**Authors:** Jian He, Jian Chen, Bohua Yu, Fuqun Wei, Weifu Liu, Peishu Huang, Songhui Wu, Xiang You, Zhisheng Chen, Ziqi Lin, Wuhua Guo, Shaowu Zhuang, Yiping Chen, Zhongwu Chen

**Affiliations:** ^1^ Department of Interventional Radiology Mengchao Hepatobiliary Hospital of Fujian Medical University Fuzhou China; ^2^ Department of Interventional Radiology, The First Affiliated Hospital Fujian Medical University Fuzhou China; ^3^ Department of Interventional Radiology, National Regional Medical Center, Binhai Campus of the First Affiliated Hospital Fujian Medical University Fuzhou China; ^4^ Department of Hepatobiliary Surgery Nanping First Hospital Affiliated to Fujian Medical University Nanping China; ^5^ Department of Oncology and Vascular Interventional Therapy Clinical Oncology School of Fujian Medical University, Fujian Cancer Hospital Fuzhou China; ^6^ Department of Radiology Jinjiang Municipal Hospital (Shanghai Sixth People's Hospital Fujian Campus) Jinjiang China; ^7^ Department of Interventional Radiology Zhangzhou Affiliated Hospital of Fujian Medical University Zhangzhou China

**Keywords:** combination therapy, HAIC, hepatocellular carcinoma, PD‐1 inhibitor, propensity score matching, TACE

## Abstract

**Background and Aims:**

The optimal sequencing of programmed cell death protein 1 (PD‐1) inhibitors relative to locoregional therapy (LRT) in unresectable hepatocellular carcinoma (uHCC) remains undefined. This study evaluated the impact of PD‐1 inhibitor timing on outcomes in patients receiving hepatic arterial infusion chemotherapy (HAIC) or transarterial chemoembolisation (TACE) plus lenvatinib and PD‐1 inhibitors.

**Methods:**

Patients with initially uHCC who received LRT in combination with lenvatinib and PD‐1 inhibitors across six academic centres were stratified by LRT modality (HAIC vs. TACE) and categorised into two groups based on PD‐1 inhibitor timing (Pre‐LRT PD‐1 and Post‐LRT PD‐1 group). Overall survival (OS) and progression‐free survival (PFS) were estimated using the Kaplan–Meier method; propensity score matching (PSM) and landmark analyses were applied to mitigate potential biases.

**Results:**

A total of 350 patients were included, with 185 in the HAIC cohort and 165 in the TACE cohort. Pre‐LRT PD‐1 group was associated with significantly improved OS in the overall population, driven primarily by the HAIC cohort rather than the TACE cohort (34.03 m vs. 30.6 m, *p* = 0.032); these findings remained consistent after PSM and landmark analyses. Subgroup analyses further supported the survival benefit of Pre‐LRT PD‐1 initiation. After PSM, Pre‐LRT PD‐1 was also associated with significantly prolonged PFS in the HAIC cohort. According to RECIST 1.1 criteria, the Pre‐LRT PD‐1 group showed a higher objective response rate (ORR) (*p* = 0.002).

**Conclusions:**

In patients with uHCC receiving HAIC combined with lenvatinib and PD‐1 inhibitors, initiation of PD‐1 inhibitors prior to HAIC is associated with superior survival outcomes.

## Summary

- This multicentre study evaluated the impact of PD‐1 inhibitor timing relative to locoregional therapy in patients with unresectable hepatocellular carcinoma (uHCC).
- Starting PD‐1 inhibitor treatment before locoregional therapy was associated with improved survival outcomes, particularly among patients receiving hepatic arterial infusion chemotherapy (HAIC).
- These findings suggest that the sequence of immunotherapy and locoregional therapy may influence treatment outcomes and provide insights for optimizing treatment strategies in uHCC.

## 1 Introduction

Hepatocellular carcinoma (HCC) continues to be one of the leading causes of cancer‐related mortality worldwide, with a significant proportion of patients presenting at an unresectable stage at diagnosis [1]. For these patients, treatment strategies have evolved rapidly from single‐modality palliative therapy to multimodal approaches that integrate systemic therapies with locoregional interventions. Notably, immune checkpoint inhibitors targeting programmed cell death protein 1 (PD‐1), particularly when used in combination with antiangiogenic agents, have revolutionised the therapeutic landscape of unresectable hepatocellular carcinoma (uHCC) [2]. Meanwhile, locoregional therapy (LRT)—including transarterial chemoembolisation (TACE) and hepatic arterial infusion chemotherapy (HAIC)—remains central to the control of intrahepatic disease [3].

The combination of PD‐1 inhibitors with LRT is supported by strong biological rationale. LRT can induce immunogenic cell death (ICD), increase the release of tumour‐associated antigens, and enhance antigen presentation, potentially sensitizing tumours to immune checkpoint blockade [4]. Conversely, PD‐1 blockade may restore the function of exhausted T cells and augment antitumour immune responses initiated by local therapy [5]. Consequently, triple therapy integrating LRT, tyrosine kinase inhibitors (TKIs), and PD‐1 inhibitors has shown promising efficacy in selected patients with uHCC [6, 7].

However, despite the increasing clinical application of these combination strategies, the optimal sequencing of PD‐1 inhibitor initiation relative to LRT remains inadequately defined. Furthermore, HAIC and TACE differ significantly in therapeutic mechanisms and biological effects. HAIC involves repeated delivery of high‐concentration regional chemotherapy, potentially leading to sustained cytotoxic stress and long‐term modulation of the tumour microenvironment [8]. In contrast, TACE primarily induces ischemic tumour necrosis through embolisation and may concurrently trigger hypoxia‐related biological alterations [9]. Given these differences, the interaction between PD‐1 inhibitor timing and treatment efficacy may not be uniform across HAIC‐ and TACE‐based regimens.

To address this clinically relevant issue, we conducted a multicentre retrospective study aiming to evaluate the association between the timing of PD‐1 inhibitor initiation and survival outcomes in patients with initially uHCC treated with LRT, lenvatinib, and PD‐1 inhibitors. We further assessed differences in the impact of treatment sequencing on prognosis between patients receiving HAIC and TACE.

## 2 Methods

### 2.1 Study Population

This multicentre retrospective cohort study enrolled consecutive patients with initially uHCC who were treated at six tertiary academic centres between January 2021 and December 2024. To be eligible for inclusion, patients had to meet the following criteria: (1) age between 18 and 80 years; (2) a diagnosis of uHCC, with unresectability determined by a multidisciplinary team (MDT) at each centre based on factors such as tumour extent, vascular invasion, presence of extrahepatic spread, liver functional reserve, anticipated future liver remnant, and overall clinical condition; (3) receipt of HAIC or TACE as the initial LRT; (4) administration of systemic therapy with lenvatinib and a PD‐1 inhibitor; (5) Child–Pugh class A or B; (6) an Eastern Cooperative Oncology Group performance status (ECOG PS) of 0 or 1; (7) at least one measurable lesion as defined by the Response Evaluation Criteria in Solid Tumours (RECIST) 1.1 criteria [10]; and (8) availability of complete baseline, treatment, and follow‐up data. Exclusion criteria included prior systemic antitumour therapy or other locoregional treatments that could confound efficacy evaluation, the presence of another active malignancy, or diagnosis of combined hepatocellular‐cholangiocarcinoma, fibrolamellar HCC, or sarcomatoid HCC. Patients were also excluded if they had Child–Pugh class C liver function, severe cardiopulmonary or renal dysfunction precluding treatment, uncontrolled infection, active gastrointestinal bleeding, or other major complications that could affect treatment delivery. Additional exclusion criteria included substantial missing data, incomplete follow‐up, or receipt of non‐protocol antitumour therapies during the initial study period. This multicentre retrospective study was conducted according to the Helsinki Declaration, received centralised approval from the ethics review committees of all participating institutions, and waived the requirement for written informed consent due to its retrospective nature.

### 2.2 Procedures

#### 2.2.1 TACE

TACE was executed using the Seldinger technique via femoral or radial arterial access. To delineate the tumour‐feeding arteries, angiography of the celiac and superior mesenteric arteries was initially performed. A microcatheter was then superselectively advanced into the tumour‐feeding branches. Through the microcatheter, an emulsion of iodised oil and chemotherapeutic agents, along with gelatin sponge particles, embolisation microspheres, or other embolic materials, was slowly infused until there was noticeable reduction in tumour staining or significant slowing or complete cessation of arterial flow. The MDT determined the necessity for repeat TACE based on tumour response, liver reserve, and treatment tolerance.

#### 2.2.2 HAIC

HAIC was conducted using a standard FOLFOX (oxaliplatin, leucovorin, fluorouracil) regimen. After arterial access was obtained via the femoral or radial artery, hepatic angiography was performed to assess tumour vascular supply. A microcatheter was positioned in the major tumour‐feeding artery to enable continuous infusion chemotherapy. The regimen included oxaliplatin at a dose of 85 mg/m^2^, leucovorin at 400 mg/m^2^, and fluorouracil at 400 mg/m^2^ administered via hepatic arterial infusion on day 1, followed by a continuous infusion of fluorouracil at 2400 mg/m^2^ over 24–46 h. HAIC was typically repeated every 3 weeks, with the MDT adjusting the treatment cycles based on tumour response, liver reserve, and adverse reactions.

#### 2.2.3 Systemic Therapy

PD‐1 inhibitors, including sintilimab, tislelizumab, and camrelizumab, were administered intravenously according to their standard approved dosing regimens, with administration intervals of every 3 weeks. In cases of intolerable treatment‐related adverse events (TRAEs), treatment delay, interruption, or permanent discontinuation was permitted. Patients were categorised into groups based on the timing of their first PD‐1 inhibitor dose relative to the first LRT session: the Pre‐LRT PD‐1 group included patients who received their first PD‐1 inhibitor dose before the first LRT, while the Post‐LRT PD‐1 group included those who initiated PD‐1 treatment after the completion of the first LRT, typically during the post‐procedural recovery period. All patients received lenvatinib therapy, which commenced after discharge following the first LRT. The initial dosing of lenvatinib was 12 mg/day for patients weighing ≥ 60 kg and 8 mg/day for those weighing < 60 kg. For patients experiencing intolerable TRAEs, lenvatinib dosing could be interrupted, reduced, or discontinued.

### 2.3 Assessment and Follow‐Up

Patient data were extracted uniformly from medical records across the six participating centres, capturing key information such as sex, age, ECOG PS, liver function grade, maximum tumour diameter, tumour number, vascular invasion, extrahepatic spread, Barcelona Clinic Liver Cancer (BCLC) stage, alpha‐fetoprotein (AFP) levels, protein induced by vitamin K absence or antagonist‐II (PIVKA‐II), along with haematological and biochemical parameters. The extent of portal vein tumour thrombus (PVTT) was classified according to the classification of PVTT by the Liver Cancer Study Group of Japan [11]. Chest CT and contrast‐enhanced liver CT or MRI were performed every 6–8 weeks, with positron emission tomography (PET‐CT) conducted when clinically indicated.

The primary endpoints of the study were overall survival (OS) and progression‐free survival (PFS). OS was defined as the time from the initiation of the first therapy to the date of death from any cause or the last follow‐up, while PFS was measured from the start of the first therapy to tumour progression or death. Secondary endpoints included the objective response rate (ORR), disease control rate (DCR), and TRAEs. Tumour response was evaluated based on findings from enhanced CT or MRI, according to both the modified Response Evaluation Criteria in Solid Tumours (mRECIST) [12] and RECIST 1.1 criteria [10]. The ORR was defined as the proportion of patients achieving a complete response (CR) or partial response (PR), whereas DCR was defined as the sum of CR, PR, and stable disease (SD). TRAEs were assessed using the National Cancer Institute's Common Terminology Criteria for Adverse Events (CTCAE), version 5.0 [13].

### 2.4 Statistical Analysis

Continuous variables are expressed as median (interquartile range), while categorical variables are presented as counts (percentages). For between‐group comparisons, Student's *t*‐test or the Mann–Whitney *U* test was used for continuous variables, and the chi‐square test or Fisher's exact test was applied for categorical variables. OS and PFS were estimated using the Kaplan–Meier (KM) method, with comparisons made using the log‐rank test. Univariable and multivariable Cox proportional hazards models were employed to identify factors associated with OS and PFS. Variables with a *p*‐value < 0.10 in univariable analysis, as well as covariates with clinical relevance, were included in the multivariable models.

To address baseline imbalances, propensity score matching (PSM) was performed separately for the HAIC and TACE cohorts (Table S1). A logistic regression model incorporating age, sex, ECOG performance status, HBV infection status, HCV infection status, HBV‐DNA level, AFP level, Child–Pugh class, cirrhosis status, total bilirubin, tumour size, tumour number, PVTT grade, EHS, PD‐1 inhibitor type, and the number of LRT was used to calculate propensity scores. We applied 1:1 nearest‐neighbour matching without replacement, with a calliper width of 0.2. The balance of baseline characteristics between matched groups was evaluated using standardised mean differences (SMD), where an absolute SMD < 0.2 for all covariates after matching was defined as adequate balance [14, 15]. To minimise the impact of time‐dependent and immortal time bias related to treatment sequencing, we performed landmark analyses at 3, 6, 12, 18 and 24 months. All statistical tests were two‐sided, with a *p*‐value of less than 0.05 considered statistically significant. Statistical analyses were conducted using Rstudio (version 4.2.2).

## 3 Results

### 3.1 Baseline Patient Characteristics

A total of 350 patients were included, comprising 185 patients treated with HAIC and 165 treated with TACE (Table 1). The two cohorts were generally comparable with respect to age, sex, and most baseline laboratory parameters. However, patients in the HAIC cohort had significantly poorer liver function, as reflected by higher TBIL levels, prolonged prothrombin time, and lower ALB levels (all *p* < 0.01). Consistently, ALBI Grade 2 was more frequent in the HAIC cohort, whereas ALBI Grade 1 was more common in the TACE cohort (*p* = 0.003). In terms of tumour burden, patients in the HAIC cohort presented with more advanced disease, including a higher proportion of large tumours (≥ 10 cm), multiple tumours, and more extensive macrovascular invasion (all *p* < 0.05). Accordingly, advanced disease stage was more common in the HAIC cohort based on the Barcelona Clinic Liver Cancer (BCLC) staging system (*p* < 0.001). The distribution of PD‐1 inhibitor timing was similar between the two cohorts (*p* = 0.2).

**TABLE 1:** Baseline characteristics.

Characteristic	Overall *N* = 350	HAIC *N* = 185	TACE *N* = 165	*p* ^a^
Age, *n* (%)				0.3
< 60	212 (61%)	117 (63%)	95 (58%)	
≥ 60	138 (39%)	68 (37%)	70 (42%)	
Gender, *n* (%)				> 0.9
Male	322 (92%)	170 (92%)	152 (92%)	
Female	28 (8.0%)	15 (8.1%)	13 (7.9%)	
ECOG, *n* (%)				**0.012**
0	285 (81%)	141 (76%)	144 (87%)	
1	65 (19%)	44 (24%)	21 (13%)	
WBC (×10^9^/L), Median (Q1, Q3)	6.30 (4.86, 8.02)	6.30 (4.83, 8.05)	6.33 (5.04, 7.97)	> 0.9
Hb (g/L), Median (Q1, Q3)	134 (120, 148)	130 (119, 145)	137 (125, 149)	**0.031**
PLT (×10^9^/L), Median (Q1, Q3)	208 (147, 265)	208 (145, 276)	212 (153, 257)	0.9
Tbil (μmol/L), Median (Q1, Q3)	14 (10, 20)	16 (11, 21)	13 (9, 19)	**0.004**
ALB (g/L), Median (Q1, Q3)	38.5 (35.0, 41.4)	37.2 (34.0, 40.6)	40.0 (36.2, 42.8)	**< 0.001**
ALT (U/L), Median (Q1, Q3)	40 (26, 61)	42 (30, 64)	37 (23, 56)	**0.028**
AST (U/L), Median (Q1, Q3)	59 (39, 97)	64 (47, 108)	48 (33, 75)	**< 0.001**
GGT (U/L), Median (Q1, Q3)	157 (90, 275)	197 (124, 323)	116 (69, 188)	**< 0.001**
PT (s), Median (Q1, Q3)	13.10 (12.10, 13.80)	13.30 (12.30, 13.90)	12.70 (12.00, 13.50)	**< 0.001**
HBV_infection, *n* (%)				**0.007**
Negative	69 (20%)	47 (25%)	22 (13%)	
Positive	281 (80%)	138 (75%)	143 (87%)	
HCV_infection, *n* (%)				> 0.9
Negative	344 (98%)	182 (98%)	162 (98%)	
Positive	6 (1.7%)	3 (1.6%)	3 (1.8%)	
HBV_DNA copies, *n* (%)				0.6
≤ 500 IU/mL	130 (37%)	66 (36%)	64 (39%)	
> 500 IU/mL	220 (63%)	119 (64%)	101 (61%)	
AFP, *n* (%)				0.7
< 400 ng/mL	160 (46%)	82 (44%)	78 (47%)	
≥ 400 ng/mL	190 (54%)	103 (56%)	87 (53%)	
DCP (mAU/mL), Median (Q1, Q3)	6156 (561, 29 445)	7878 (1033, 30 000)	3869 (299, 25 484)	0.094
Cirrhosis, *n* (%)	261 (75%)	129 (70%)	132 (80%)	**0.038**
Child‐Pugh class, *n* (%)				0.6
Child A	294 (84%)	153 (83%)	141 (85%)	
Child B	56 (16%)	32 (17%)	24 (15%)	
ALBI_grade, *n* (%)				**0.003**
ALBI 1	241 (69%)	113 (61%)	128 (78%)	
ALBI 2	103 (29%)	69 (37%)	34 (21%)	
ALBI 3	6 (1.7%)	3 (1.6%)	3 (1.8%)	
Tumour size, *n* (%)				**0.002**
< 10 cm	131 (37%)	55 (30%)	76 (46%)	
≥ 10 cm	219 (63%)	130 (70%)	89 (54%)	
Tumour number, *n* (%)				**0.010**
Single	89 (25%)	36 (19%)	53 (32%)	
Multiple	261 (75%)	149 (81%)	112 (68%)	
PVTT, *n* (%)				**< 0.001**
VP0	126 (36%)	35 (19%)	91 (55%)	
VP2	50 (14%)	27 (15%)	23 (14%)	
VP3	108 (31%)	69 (37%)	39 (24%)	
VP4	66 (19%)	54 (29%)	12 (7.3%)	
EHS, *n* (%)	99 (28%)	61 (33%)	38 (23%)	0.052
BCLC_stage, *n* (%)				**< 0.001**
A	19 (5.4%)	4 (2.2%)	15 (9.1%)	
B	89 (25%)	27 (15%)	62 (38%)	
C	242 (69%)	154 (83%)	88 (53%)	
LRT_number, *n* (%)				0.10
< 3	138 (39%)	65 (35%)	73 (44%)	
≥ 3	212 (61%)	120 (65%)	92 (56%)	
PD_1 type, *n* (%)				**0.044**
Camrelizumab	163 (47%)	91 (49%)	72 (44%)	
Tislelizumab	124 (35%)	55 (30%)	69 (42%)	
Sintilimab	63 (18%)	39 (21%)	24 (15%)	
PD1_timing, *n* (%)				0.2
Pre‐LRT PD1	128 (37%)	61 (33%)	67 (41%)	
Post‐LRT PD1	222 (63%)	124 (67%)	98 (59%)	
PD1_LRT_interval time (days), Median (Q1, Q3)				
Pre‐LRT PD1	−2.00 (−1.00, −4.00)	−3.00 (−1.00, −5.00)	−1.00 (−1.00, −2.00)	0.667
Post‐LRT PD1	6.00 (3.00, 17.00)	5.00 (3.00, 10.00)	8.00 (2.75, 31.00)	

*Note:* The bold marking is implemented to highlight the parts corresponding to *p* < 0.05.

Abbreviations: AFP, alpha‐Fetoprotein; DCP, des‐gamma‐carboxy prothrombin; EHS, extrahepatic spread; LRT locoregional therapy; PVTT, portal vein tumour thrombus.

^a^ Pearson's Chi‐squared test; Wilcoxon rank sum test.

### 3.2 Tumour Response

**FIGURE 1:**
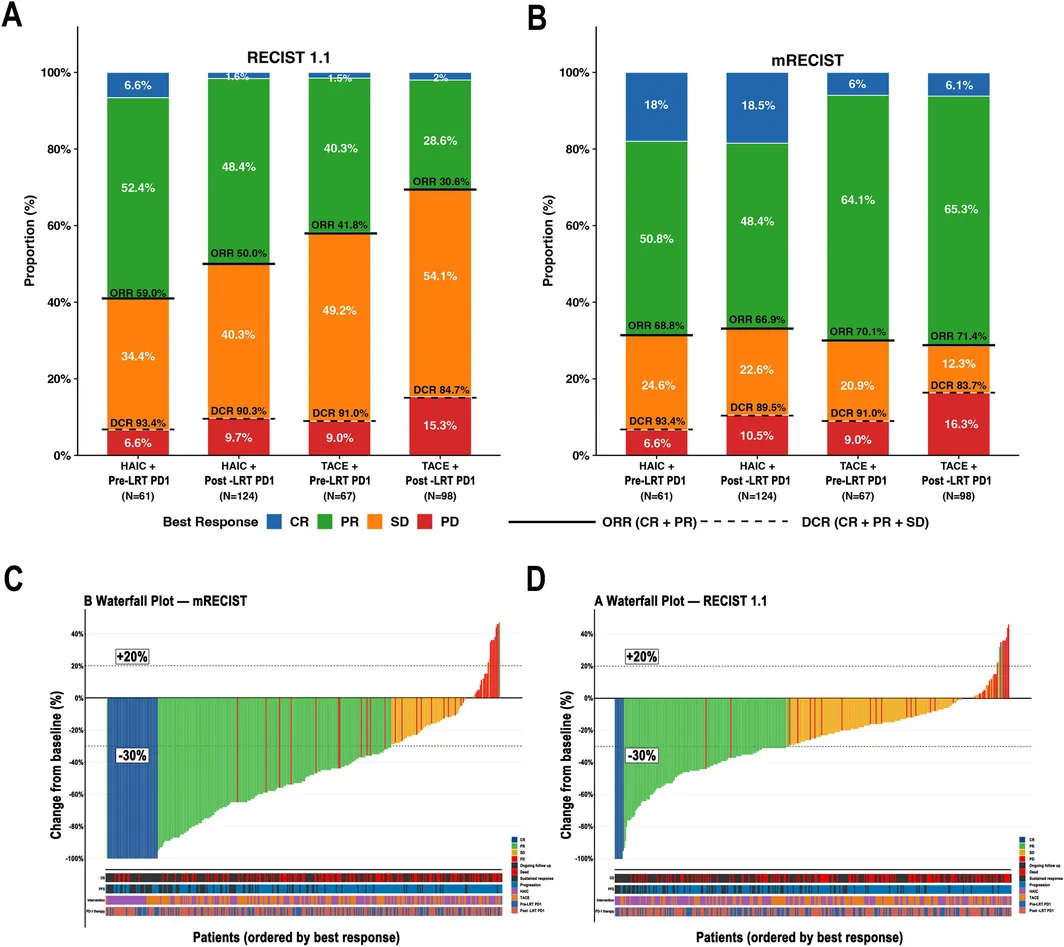
Tumour response assessment by RECIST 1.1 and mRECIST in HCC patients receiving different treatment modalities. (A, B) Stacked bar charts showing the distribution of best overall response according to RECIST 1.1 (A) and mRECIST (B) across four treatment subgroups. (C, D) Waterfall plots illustrating the maximum percentage change in target lesion size from baseline, as evaluated by RECIST 1.1 (C) and mRECIST (D).

Radiological tumour response was evaluated according to both RECIST 1.1 and mRECIST criteria (Figure 1). According to RECIST 1.1 criteria, the distribution of best tumour response differed significantly among the four treatment sequence groups (*p* = 0.002), with a higher ORR observed in the Pre‐LRT PD‐1 groups. In the HAIC cohort, the Pre‐LRT PD‐1 group showed a numerically higher ORR (59.0% vs. 50.0%) and DCR (93.4% vs. 90.3%) compared with the Post‐LRT PD‐1 group. Similarly, in the TACE cohort, the Pre‐LRT PD‐1 group demonstrated higher ORR (41.8% vs. 30.6%) and DCR (91.0% vs. 84.7%) compared with the Post‐LRT PD‐1 group. According to mRECIST criteria, no statistically significant differences in tumour response outcomes were observed among the four treatment sequence groups. In the HAIC cohort, the ORR was 68.8% in the Pre‐LRT PD‐1 group and 66.9% in the Post‐LRT PD‐1 group, while the DCR was 93.4% and 89.5%, respectively. In the TACE cohort, the ORR was comparable between the Pre‐LRT and Post‐LRT PD‐1 groups (70.1% vs. 71.4%), whereas the Pre‐LRT PD‐1 group showed a numerically higher DCR (91.0% vs. 83.7%) (Table S2).

### 3.3 Survival Analysis

**FIGURE 2:**
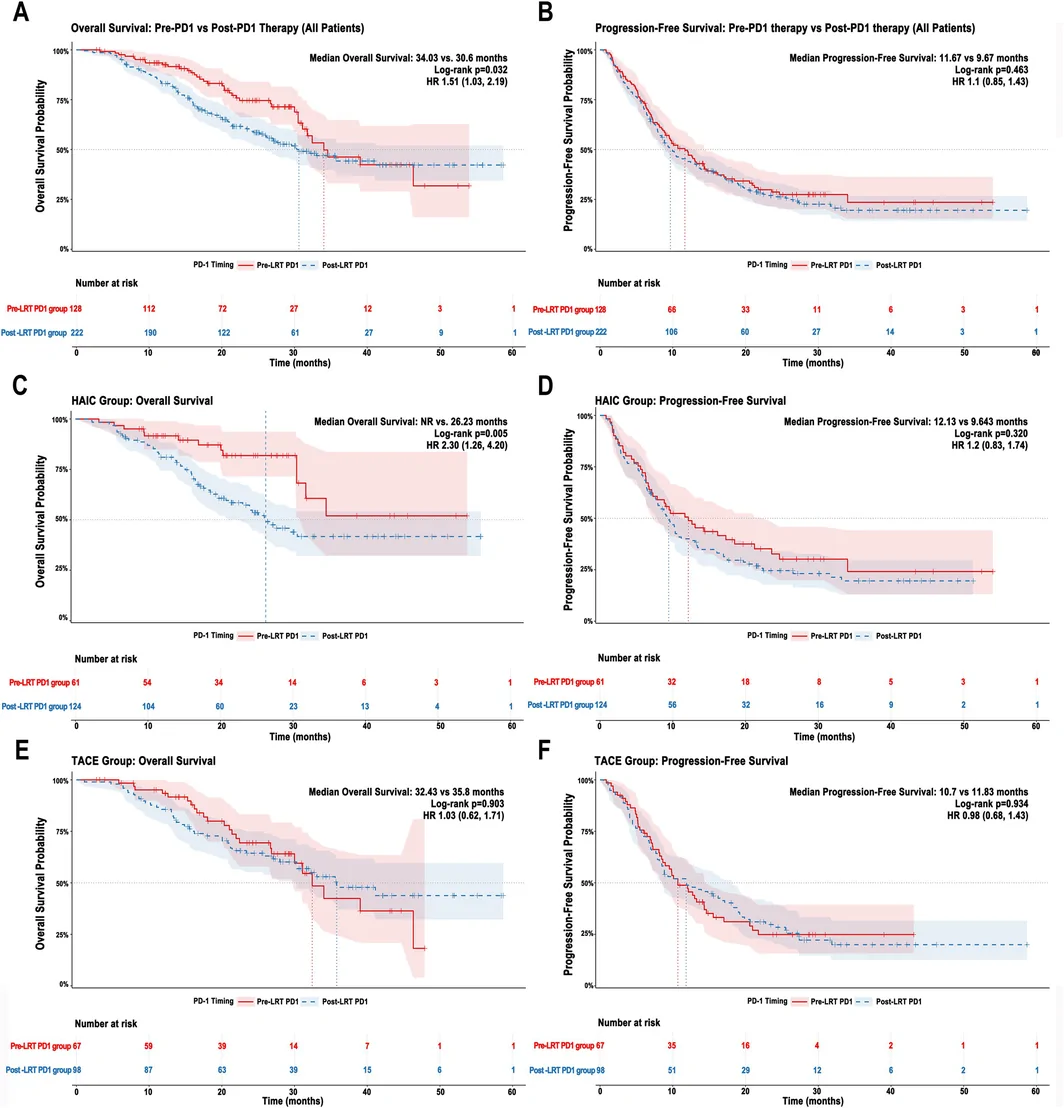
Survival outcomes of patients treated with LRT plus lenvatinib and PD‐1 inhibitors. (A, B) Kaplan–Meier curves comparing OS (A) and PFS (B) in all patients. (C, D) Kaplan–Meier curves comparing OS (C) and PFS (D) within the HAIC subgroup. (E, F) Kaplan–Meier curves comparing OS (E) and PFS (F) within the TACE subgroup.

The median follow‐up time was 21.0 (IQR, 13.8–29.9) months. In the overall cohort, median OS was 34.03 and 30.6 months in the Pre‐LRT and Post‐LRT PD‐1 groups, respectively (HR = 1.51, 95% CI = 0.28–0.70, *p* = 0.032; Figure 2A). Median PFS was 11.67 and 9.67 months, respectively (HR = 1.10, 95% CI = 0.85–1.43, *p* = 0.463; Figure 2B). When stratified by intervention modality, in the HAIC cohort, median OS was not reached and 26.23 months in the Pre‐LRT and Post‐LRT PD‐1 groups, respectively (HR = 2.30, 95% CI = 1.26–4.20, *p* = 0.005; Figure 2C), whereas median PFS was 12.13 and 9.43 months (HR = 1.20, 95% CI = 0.83–1.74, *p* = 0.320; Figure 2D). In the TACE cohort, median OS was 32.43 and 35.8 months (HR = 1.03, 95% CI = 0.62–1.71, *p* = 0.903; Figure 2E), and median PFS was 10.7 and 11.8 months (HR = 0.98, 95% CI = 0.68–1.43, *p* = 0.934; Figure 2F), indicating no significant differences between the two groups.

### 3.4 Prognostic Factor for OS and PFS

For OS, Post‐LRT PD‐1 sequencing (HR = 1.51; 95% CI, 1.03–2.19; *p* = 0.034), multiple tumours (HR = 1.52; 95% CI, 1.01–2.29; *p* = 0.046), PVTT stage VP3 (HR = 1.95; 95% CI, 1.27–3.00; *p* = 0.002) and VP4 (HR = 2.10; 95% CI, 1.29–3.41; *p* = 0.003), extrahepatic spread (HR = 2.11; 95% CI, 1.51–2.97; *p* < 0.001), and BCLC stage C (HR = 2.78; 95% CI, 1.13–6.82; *p* = 0.026) were associated with increased risk of death in univariable analyses. In multivariable analysis, EHS (HR = 1.58; 95% CI, 1.06–2.35; *p* = 0.024) and multiple tumours (HR = 1.71; 95% CI, 1.07–2.73; *p* = 0.026) remained an independent risk factor for mortality. For PFS, female (HR = 1.83; 95% CI, 1.15–2.91; *p* = 0.011), lower albumin (ALB) levels (HR = 0.96; 95% CI, 0.92–1.00; *p* = 0.033), and multiple tumours (HR = 1.57; 95% CI, 1.11–2.23; *p* = 0.011) were independent risk factors for disease progression. Detailed results are presented in Table 2.

**TABLE 2:** Univariable and Multivariable cox regression analysis for OS and PFS.

Characteristic	OS						PFS					
	Univariable analysis			Multivariable analysis			Univariable analysis			Multivariable analysis		
	HR	95% CI	*p*	HR	95% CI	*p*	HR	95% CI	*p*	HR	95% CI	*p*
Age												
< 60 years	—	—		—	—		—	—		—	—	
≥ 60 years	0.90	0.63, 1.27	0.5	0.92	0.64, 1.32	0.6	0.85	0.66, 1.10	0.2	0.80	0.61, 1.05	0.10
Gender												
Male	—	—		—	—		—	—		—	—	
Female	1.43	0.81, 2.54	0.2	1.51	0.82, 2.80	0.2	1.52	0.99, 2.31	0.053	1.83	1.15, 2.91	**0.011**
ECOG												
0	—	—		—	—		—	—		—	—	
1	1.69	1.13, 2.53	**0.010**	1.57	0.80, 3.08	0.2	1.35	0.99, 1.84	0.055	1.15	0.72, 1.85	0.6
WBC, ×10^9^/L	1.04	0.97, 1.12	0.2				1.06	1.00, 1.11	**0.037**	1.05	0.99, 1.10	0.10
Hb, g/L	1.00	0.99, 1.00	0.3				0.99	0.99, 1.00	**0.049**	1.00	0.99, 1.00	0.5
PT, s	1.05	0.92, 1.20	0.5				0.92	0.83, 1.02	0.11			
PLT, ×10^9^/L	1.00	1.00, 1.00	> 0.9				1.00	1.00, 1.00	0.3			
Tbil, umol/L	0.99	0.98, 1.01	0.3				1.00	0.99, 1.01	> 0.9			
ALB, g/L	0.98	0.95, 1.01	0.2				0.97	0.95, 0.99	**0.012**	0.96	0.92, 1.00	**0.033**
ALT, U/L	1.00	0.99, 1.00	0.13				1.00	1.00, 1.00	0.3			
AST, U/L	1.00	1.00, 1.00	0.4				1.00	1.00, 1.00	0.2			
GGT, U/L	1.00	1.00, 1.00	0.8				1.00	1.00, 1.00	0.4			
ALBI_grade												
ALBI 1	—	—		—	—		—	—		—	—	
ALBI 2	1.20	0.84, 1.71	0.3	0.77	0.42, 1.41	0.4	1.26	0.97, 1.64	0.089	0.75	0.45, 1.24	0.3
ALBI 3	0.44	0.06, 3.16	0.4	0.43	0.06, 3.25	0.4	0.69	0.22, 2.17	0.5	0.25	0.06, 1.02	0.053
Child‐Pugh class												
Child A	—	—					—	—				
Child B	1.17	0.75, 1.83	0.5				1.22	0.88, 1.69	0.2			
AFP												
< 400 ng/mL	—	—		—	—		—	—		—	—	
≥ 400 ng/mL	1.21	0.86, 1.69	0.3	1.06	0.75, 1.51	0.7	0.88	0.69, 1.13	0.3	0.83	0.64, 1.08	0.2
HBV_infection												
Negative	—	—					—	—				
Positive	0.78	0.53, 1.17	0.2				1.37	0.99, 1.89	0.061			
HBV_DNA												
≤ 500 IU/mL	—	—					—	—				
> 500 IU/mL	0.83	0.59, 1.17	0.3				1.08	0.83, 1.40	0.6			
Tumour size												
< 10 cm	—	—		—	—		—	—		—	—	
≥ 10 cm	1.14	0.81, 1.61	0.5	0.90	0.61, 1.33	0.6	1.27	0.98, 1.64	0.074	1.24	0.93, 1.66	0.14
Tumour number												
Single	—	—		—	—		—	—		—	—	
Multiple	1.52	1.01, 2.29	**0.046**	1.71	1.07, 2.73	**0.026**	1.46	1.09, 1.97	**0.012**	1.57	1.11, 2.23	**0.011**
PVTT												
VP0	—	—		—	—		—	—		—	—	
VP2	1.55	0.89, 2.68	0.12	1.11	0.55, 2.27	0.8	1.14	0.77, 1.68	0.5	0.83	0.48, 1.43	0.5
VP3	1.95	1.27, 3.00	**0.002**	1.39	0.75, 2.56	0.3	1.05	0.78, 1.43	0.7	0.65	0.41, 1.04	0.074
VP4	2.10	1.29, 3.41	**0.003**	1.71	0.82, 3.55	0.2	1.30	0.92, 1.84	0.13	0.96	0.56, 1.66	0.9
EHS												
No	—	—		—	—		—	—		—	—	
Yes	2.11	1.51, 2.97	**< 0.001**	1.58	1.06, 2.35	**0.024**	1.33	1.02, 1.73	**0.035**	0.98	0.72, 1.34	0.9
BCLC_stage												
A	—	—		—	—		—	—		—	—	
B	1.02	0.38, 2.75	> 0.9	0.58	0.20, 1.69	0.3	1.38	0.72, 2.62	0.3	0.82	0.40, 1.70	0.6
C	2.78	1.13, 6.82	**0.026**	1.20	0.40, 3.57	0.7	1.74	0.95, 3.20	0.075	1.24	0.57, 2.69	0.6
LRT_number												
< 3	—	—		—	—		—	—		—	—	
≥ 3	1.23	0.87, 1.75	0.2	1.01	0.70, 1.46	> 0.9	1.08	0.84, 1.39	0.6	1.08	0.83, 1.41	0.6
Intervention_mode												
HAIC	—	—		—	—		—	—		—	—	
TACE	0.90	0.64, 1.25	0.5	1.33	0.88, 2.01	0.2	0.94	0.73, 1.20	0.6	1.14	0.85, 1.53	0.4
PD_1type												
Camrelizumab	—	—		—	—		—	—		—	—	
Tislelizumab	1.03	0.72, 1.48	0.9	1.05	0.71, 1.56	0.8	0.94	0.71, 1.24	0.6	1.04	0.77, 1.39	0.8
Sintilimab	0.62	0.36, 1.07	0.089	0.58	0.32, 1.03	0.063	1.19	0.85, 1.67	0.3	1.20	0.84, 1.71	0.3
PD1_timing												
Pre‐LRT PD1	—	—		—	—		—	—		—	—	
Post‐LRT PD1	1.51	1.03, 2.19	**0.034**	1.35	0.91, 2.02	0.14	1.10	0.85, 1.43	0.5	1.07	0.80, 1.41	0.7

*Note:* The bold marking is implemented to highlight the parts corresponding to *p* < 0.05.

Abbreviations: AFP, alpha‐fetoprotein; CI, confidence interval; EHS, extrahepatic spread; HR, hazard ratio; OS, overall survival; PFS progression‐free survival; PVTT, portal vein tumour thrombus.

### 3.5 Interaction Between LRT Modality and PD‐1 Timing

To evaluate whether the association between PD‐1 timing and survival outcomes differed according to LRT modality, multivariable Cox models incorporating an interaction term between intervention modality and PD‐1 timing were performed. A significant interaction was observed for OS (interaction *p* = 0.030), whereas no significant interaction was detected for PFS (interaction *p* = 0.167). In the interaction model, the unfavourable association of Post‐LRT PD‐1 therapy observed in the HAIC cohort was attenuated among patients receiving TACE (interaction HR = 0.41, 95% CI: 0.18–0.93, *p* = 0.033) (Table S3).

### 3.6 Subgroup Analysis

Subgroup analyses were performed using Cox proportional hazards models to assess the consistency of treatment effects on PFS and OS for Post‐LRT PD‐1 versus Pre‐LRT PD‐1 therapy (Figure S1). The overall HR for PFS was 1.10 (95% CI: 0.85–1.43; *p* = 0.462), indicating no significant difference between the treatment groups. No significant interaction effects were observed for PFS across most clinical variables, including age, gender, AFP level, hepatitis B virus (HBV) infection status, Child–Pugh (CP) class, ALBI grade, tumour size, tumour number, macrovascular invasion, extrahepatic spread, BCLC stage, and treatment modality (all *p* > 0.05). For OS, the overall HR was 1.51 (95% CI: 1.03–2.19; *p* = 0.034), demonstrating significantly superior survival with Pre‐LRT PD‐1 therapy. The subgroup analyses for OS revealed several significant interactions: among patients without HBV infection, Post‐LRT PD‐1 therapy was associated with worse OS compared with Pre‐LRT PD‐1 therapy (HR = 3.49, 95% CI: 1.34–9.08; *p* = 0.01). Post‐LRT PD‐1 therapy was associated with worse OS among patients with ALBI Grade 1 (HR = 1.68, 95% CI: 1.05–2.69; *p* = 0.032). Inferior OS with Post‐LRT PD‐1 therapy was also consistently observed for patients with tumour size ≥ 10 cm, multiple tumours, PVTT stage VP3, BCLC stage C disease, PD‐1 inhibitor use specifically of tislelizumab, and treated with HAIC (all *p* < 0.05).

### 3.7 Sensitivity Analysis

**FIGURE 3:**
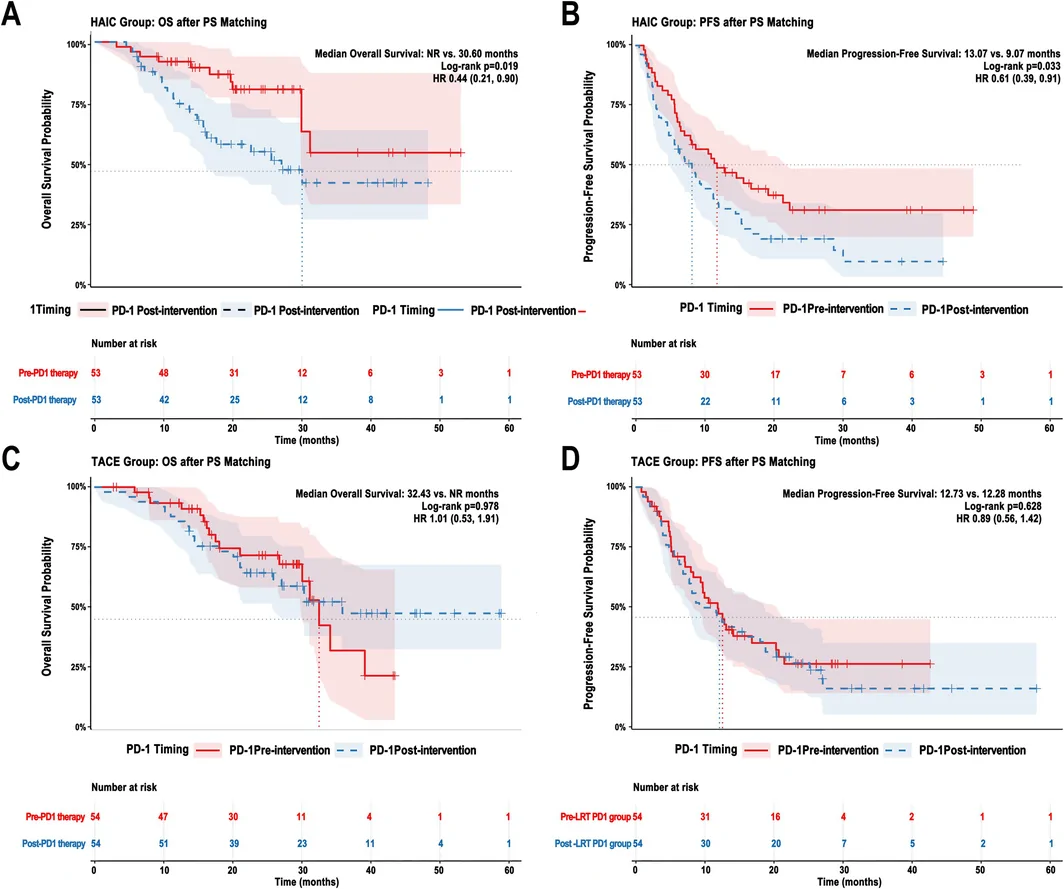
Survival outcomes of patients receiving LRT combined with lenvatinib and PD‐1 inhibitors after PSM. (A, B) Kaplan–Meier curves for OS (A) and PFS (B) in the HAIC subgroup after PSM. (C, D) Kaplan–Meier curves for OS (C) and PFS (D) in the TACE subgroup after PSM.

After PSM, in the HAIC cohort, median OS was not reached and 30.60 months in the Pre‐LRT and Post‐LRT PD‐1 groups, respectively (HR = 0.44, 95% CI = 0.21–0.90, *p* = 0.019; Figure 3A), and median PFS was 13.07 and 9.07 months (HR = 0.61, 95% CI = 0.39–0.91, *p* = 0.033; Figure 3B). In the TACE cohort, median OS was 32.43 months and not reached (HR = 1.01, 95% CI = 0.53–1.91, *p* = 0.978; Figure 3C), and median PFS was 12.73 and 12.28 months (HR = 0.89, 95% CI = 0.56–1.42, *p* = 0.628; Figure 3D), indicating no significant differences between groups.

In landmark analyses performed at 3‐, 6‐, 12‐, 18‐, and 24‐month time points to further assess the robustness of the findings (Figure S2A–E), similar survival trends were observed across different landmark windows. In the HAIC cohort, Pre‐LRT PD‐1 therapy consistently showed superior OS compared with Post‐LRT PD‐1 therapy. At the 3‐month landmark analysis, median OS was not reached versus 23.23 months (HR = 0.45, 95% CI: 0.25–0.82, *p* = 0.008), while the PFS difference was not statistically significant (HR = 0.73, 95% CI: 0.48–1.11, *p* = 0.139). Similar OS patterns were observed in the 6‐, 12‐, 18‐, and 24‐month landmark analyses. In contrast, no significant differences in OS or PFS were observed between PD‐1 timing groups in the TACE cohort across all landmark analyses.

In addition, the distribution of distinct PD‐1 inhibitors varied across study groups (Table S4). We further performed sensitivity analyses stratified by individual PD‐1 agent (camrelizumab, tislelizumab, and sintilimab) to evaluate whether the observed association between PD‐1 sequencing and survival outcomes was modified by specific drug subtypes (Table S5). In the HAIC cohort, the adverse association of Post‐LRT PD‐1 therapy was significant only among patients receiving tislelizumab, both for OS (HR = 3.61, 95% CI: 1.23–10.57, *p* = 0.019) and PFS (HR = 2.52, 95% CI: 1.22–5.17, *p* = 0.012). No significant survival differences associated with PD‐1 timing were observed in the camrelizumab or sintilimab subgroups (all *p* > 0.05). In the TACE cohort, no significant differences in OS or PFS between Pre‐LRT and Post‐LRT PD‐1 strategies were observed across the three PD‐1 inhibitor subgroups, although the tislelizumab subgroup showed a borderline increased risk for OS (HR = 3.31, 95% CI: 0.99–11.14, *p* = 0.052).

### 3.8 Safety

The combination therapies were well tolerated, and no treatment‐related deaths occurred. Toxicity profiles aligned with the expected effects of each locoregional modality (Table 3). Although Grade 3 or 4 TRAEs arose in both cohorts, they were manageable through dose modifications or supportive care. In the TACE cohort, Grade 3 or 4 abdominal pain (10.9% vs. 2.2%; *p* = 0.001) and elevated AST (15.2% vs. 8.1%; *p* = 0.046) were significantly more frequent, reflecting postembolisation syndrome. The HAIC cohort, conversely, showed higher rates of any‐grade nausea/vomiting (61.6% vs. 35.2%; *p* < 0.001) and Grade 3 or 4 neutropenia (11.9% vs. 2.4%; *p* = 0.001). Systemic toxicities attributable to lenvatinib and PD‐1 inhibitors—such as hypertension (Grade 3/4: 8.5% vs. 8.1%), Hand–foot skin reaction (27.9% vs. 27.6%), proteinuria (23.0% vs. 22.7%) and immune‐related hepatitis (3.6% vs. 7.6%)—were comparable between the groups (all *p* > 0.05). Detailed comparisons of TRAEs stratified by PD‐1 sequencing (Pre‐LRT vs. Post‐LRT PD‐1) across HAIC and TACE subgroups are provided in Table S6.

**TABLE 3:** Treatment‐related adverse events.

Adverse event	Any grade			Grade 3/4		
	TACE + Len + PD‐1 (*n* = 165)	HAIC + Len + PD‐1 (*n* = 185)	*p* * (any grade)	TACE + Len + PD‐1 (*n* = 165)	HAIC + Len + PD‐1 (*n* = 185)	*p* * (Grade 3/4)
Abdominal pain	115 (69.7%)	46 (24.9%)	**< 0.001**	18 (10.9%)	4 (2.2%)	**0.001**
Fever	92 (55.8%)	37 (20.0%)	**< 0.001**	3 (1.8%)	0 (0.0%)	0.094
Nausea and vomiting	58 (35.2%)	114 (61.6%)	**< 0.001**	5 (3.0%)	12 (6.5%)	0.147
Diarrhoea	45 (27.3%)	52 (28.1%)	0.865	3 (1.8%)	4 (2.2%)	0.990
Decreased appetite	61 (37.0%)	78 (42.2%)	0.316	2 (1.2%)	4 (2.2%)	0.686
Elevated AST	124 (75.2%)	102 (55.1%)	**< 0.001**	25 (15.2%)	15 (8.1%)	**0.046**
Elevated ALT	110 (66.7%)	89 (48.1%)	**0.001**	18 (10.9%)	11 (5.9%)	0.108
Hyperbilirubinaemia	53 (32.1%)	46 (24.9%)	0.134	6 (3.6%)	4 (2.2%)	0.528
Hypoalbuminaemia	41 (24.8%)	55 (29.7%)	0.301	2 (1.2%)	3 (1.6%)	0.990
Neutropenia	28 (17.0%)	83 (44.9%)	**< 0.001**	4 (2.4%)	22 (11.9%)	**0.001**
Thrombocytopenia	74 (44.8%)	96 (51.9%)	0.183	11 (6.7%)	18 (9.7%)	0.334
Anaemia	35 (21.2%)	54 (29.2%)	0.087	2 (1.2%)	6 (3.2%)	0.286
Hypertension	69 (41.8%)	76 (41.1%)	0.887	14 (8.5%)	15 (8.1%)	0.990
Fatigue	55 (33.3%)	74 (40.0%)	0.192	3 (1.8%)	5 (2.7%)	0.725
Proteinuria	38 (23.0%)	42 (22.7%)	0.944	4 (2.4%)	3 (1.6%)	0.711
Hypothyroidism	28 (17.0%)	33 (17.8%)	0.835	0 (0.0%)	0 (0.0%)	—
Rash	21 (12.7%)	26 (14.1%)	0.707	2 (1.2%)	1 (0.5%)	0.603
Hand–foot skin reaction	46 (27.9%)	51 (27.6%)	0.947	5 (3.0%)	4 (2.2%)	0.742
Immune‐related hepatitis	6 (3.6%)	14 (7.6%)	0.248	2 (1.2%)	4 (2.2%)	0.720

*Note:* The bold marking is implemented to highlight the parts corresponding to *p* < 0.05.

* *p* values were calculated using a two‐sided *χ* ^2^ test.

## 4 Discussion

In this multicentre retrospective study, we investigated the impact of PD‐1 inhibitor timing relative to LRT in patients with uHCC treated with triple therapy. Our findings reveal that the Pre‐LRT PD‐1 sequencing strategy yields superior oncological outcomes compared to the Post‐LRT PD‐1 strategy. Specifically, in terms of tumour response, patients in the Pre‐LRT PD‐1 group demonstrated significantly higher ORR and DCR across both the TACE and HAIC cohorts according to RECIST 1.1 and mRECIST criteria. Crucially, after PSM to minimise confounding bias, this improved immediate response translated into a robust survival advantage—both in PFS and OS—predominantly within the HAIC cohort. These results suggest that upfront systemic immunotherapy acts as a critical sensitiser for subsequent locoregional interventions, particularly in HAIC‐based regimens.

The observed superiority of the Pre‐LRT PD‐1 strategy can be attributed to the concept of ‘neoadjuvant‐like immunological priming’ [16, 17]. By administering PD‐1 inhibitors prior to locoregional disruption, the therapy effectively reinvigorates exhausted CD8+ T cells and normalises the tumour immune microenvironment (TIME) before the heavy distinct antigen load is released. When LRT is subsequently performed, the induction of ICD releases a cascade of tumour neoantigens. Because the immune system has already been ‘unbraked’ by the upfront PD‐1 blockade, these antigens are met with a primed, highly responsive T‐cell repertoire, facilitating a more efficient and profound anti‐tumour immune attack [18]. In addition, the administration of PD‐1 inhibitors prior to LRT may promote transient vascular normalisation [19]. This strategic sequencing ensures the systemic immune repertoire is not only quantitatively expanded but also qualitatively fortified against the localised immunosuppressive effects that follow intervention. This physiological improvement enhances the intratumoural delivery and homogenous distribution of chemotherapeutic agents during the subsequent HAIC or TACE procedure, thereby explaining the universally improved ORR and DCR observed in the Pre‐LRT PD‐1 group across both modalities. Beyond antigen release and vascular changes, the temporal advantage of PD‐1 blockade prior to LRT may also stem from the preservation of T‐cell stemness. Administering PD‐1 inhibition while the primary tumour architecture remains intact facilitates the expansion of progenitor exhausted T cells, which retain greater proliferative capacity and are less susceptible to the terminal exhaustion typically induced by the sudden antigen release following LRT [20]. Moreover, early PD‐1 blockade can proactively reprogram the myeloid compartment by favouring a shift from pro‐tumourigenic M2‐type macrophages toward anti‐tumourigenic M1‐type macrophages, thereby establishing an immune buffer that counteracts the transient influx of myeloid‐derived suppressor cells (MDSCs) recruited by TACE‐induced hypoxia [21, 22]. Importantly, the effect of PD‐1 timing may differ according to the type of locoregional therapy. HAIC and TACE induce distinct immune remodelling patterns. FOLFOX‐based HAIC has been reported to promote tertiary lymphoid structure (TLS) formation and enhance anti‐tumour immunity, potentially creating a favourable microenvironment for PD‐1 blockade [23]. In contrast, although TACE induces tumour antigen release and immunogenic cell death, it may also trigger immunosuppressive changes, including reduced CD8+ T‐cell infiltration and increased TREM2+ TAM accumulation [24]. These differences may partly explain why the benefit of Pre‐LRT PD‐1 therapy appeared more pronounced in the HAIC cohort.

However, a divergence emerges when evaluating long‐term survival. While Pre‐LRT PD‐1 sequencing improved initial tumour responses in the TACE cohort, it failed to translate into a statistically significant OS benefit, unlike in the HAIC cohort. This disparity is likely driven by the inherent biological consequences of the procedures. TACE relies on embolisation, which induces severe hypoxia and upregulates hypoxia‐inducible factor‐1 alpha (HIF‐1α) and vascular endothelial growth factor (VEGF). This hypoxic stress recruits immunosuppressive myeloid‐derived suppressor cells (MDSCs) and regulatory T cells (Tregs), creating a hostile, ‘cold’ microenvironment that eventually dampens the long‐term efficacy of the reinstated T‐cell response [25]. In contrast, HAIC provides sustained high‐concentration chemotherapy without acute arterial occlusion. This approach preserves oxygenation within the TME, thereby sustaining the immunological momentum generated by the initial Pre‐LRT PD‐1 priming. Consequently, the potent initial response in the HAIC Pre‐LRT PD‐1 group successfully translates into a durable survival tail.

Subgroup analyses further underscored the clinical necessity of the Pre‐LRT PD‐1 strategy for specific high‐risk populations. The survival benefit of upfront PD‐1 blockade was most pronounced in patients with high tumour burden (tumour size > 10 cm, multiple tumours, PVTT stage Vp3) and BCLC stage C disease. We hypothesise that these massive, biologically aggressive tumours harbour deep‐seated immunosuppression. Upfront PD‐1 blockade serves to ‘condition’ the host immune system, preventing the tolerance or T‐cell anergy that might otherwise occur if the immune system were suddenly overwhelmed by the massive antigen sink released by immediate LRT [26]. Additionally, patients with non‐viral aetiology exhibited significantly worse outcomes with the Post‐LRT PD‐1 strategy (HR = 3.49, 95% CI 1.34–9.08, *p* = 0.01). Several key caveats warrant cautious interpretation of this subgroup finding. For one thing, the non‐viral cohort only contained 69 patients, leading to limited statistical power to confirm the detected survival difference reliably. For another, all stratified subgroup analyses here were exploratory without formal multiple comparison correction, which elevates the likelihood of false‐positive results. Even with these constraints, existing literature supports that non‐viral HCC demonstrates inherently weaker responsiveness to immune checkpoint inhibitors relative to virus‐associated HCC [27], our data indicates these patients require the aggressive ‘priming’ of upfront PD‐1 blockade combined with the ICD effects of HAIC to ignite an effective immune cycle; delaying immunotherapy in this subgroup appears detrimental.

Several limitations should be acknowledged. First, due to the retrospective observational design, treatment allocation was not randomised, and residual confounding from unmeasured factors, including tumour immune microenvironment characteristics and physician treatment preference, cannot be completely excluded. Although PSM and landmark analyses were performed to reduce potential bias, prospective randomised trials are needed to validate the optimal sequencing strategy of PD‐1 inhibitors and locoregional therapies. Furthermore, incomplete archival records of lenvatinib dosing and dose adjustments across participants prevented us from conducting stratified assessments of RDI and dose modification frequencies. Second, treatment decisions, procedural details, and the choice of PD‐1 inhibitor may have varied across centres, introducing a degree of clinical heterogeneity. Third, although the overall sample size was reasonable, some subgroup analyses were based on limited numbers of patients and should therefore be interpreted cautiously. Fourth, we defined sequencing according to the relative order of first LRT and PD‐1 inhibitor initiation, but did not comprehensively model the exact treatment interval as a continuous exposure; therefore, the optimal temporal window remains uncertain. Fifth, although the overall direction of the findings was generally consistent across PD‐1 inhibitor subgroups, the adverse association of Post‐LRT PD‐1 therapy appeared more evident in patients receiving tislelizumab, particularly in the HAIC cohort. This observation may reflect potential differences in pharmacological characteristics, immune‐modulatory profiles, or patient selection patterns among different PD‐1 agents. However, these subgroup analyses were exploratory and limited by the relatively small sample sizes within individual drug categories; therefore, the possibility of chance findings cannot be excluded. Further prospective studies with larger cohorts are needed to determine whether specific PD‐1 agents modify the optimal timing of immunotherapy relative to locoregional therapy. Finally, translational biomarker data were unavailable, limiting our ability to directly investigate the immunological mechanisms underlying the observed modality‐specific sequencing effect.

## 5 Conclusion

In patients with uHCC receiving HAIC‐based triple therapy, administration of PD‐1 inhibitors prior to LRT was associated with improved tumour response, as well as significantly prolonged long‐term survival and delayed disease progression. In contrast, the prognostic impact was limited in TACE‐based triple therapy settings. These findings suggest that the sequencing of PD‐1 inhibitors according to different LRT strategies may influence treatment efficacy, providing a rationale for future prospective investigations.

## Author Contributions

Z.C., Y.C., S.Z., and W.G. conceptualized and designed the study, conducting the investigation. J.H., J.C., B.Y., F.W., W.L., P.H., S.W., X.Y., Z.C., and Z.L. collected the data. J.H., J.C., and B.Y. analysed the data. J.H., J.C., B.Y. and F.W. wrote the original draft. Z.C., Y.C., and W.G. have accessed and verified the data. Z.C. and Y.C. provided supervision. All authors reviewed and approved the final version of the manuscript. All authors had full access to all the data in the study and had final responsibility for the decision to submit for publication.

## Funding

This study was funded by the Joint Funds for the Innovation of Science and Technology, Fujian Province (No. 2024Y9126 and No. 2024Y9166), and University‐Industry Research Cooperation Project of Science and Technology, Fujian province (No. 2024Y4005). The Provincial Natural Science Foundation of Fujian (2023J011463).

## Ethics Statement

All procedures performed in studies involving human participants were in accordance with the ethical standards of the institutional and/or national research committee, and this research was conducted ethically in accordance with the World Medical Association Declaration of Helsinki.

## Conflicts of Interest

The authors declare no conflicts of interest.

## Supporting information


**Figure S1:** Subgroup analyses of PFS (A) and OS (B) according to PD‐1 inhibitor timing in all patients. AFP, alpha‐fetoprotein; EHS, extrahepatic spread; HAIC, hepatic arterial infusion chemotherapy; PSM, propensity score matching; PVTT, portal vein tumour thrombus; TACE, transarterial chemoembolisation.
**Figure S2:** Landmark survival analysis in the HAIC and TACE subgroups. (A) 3‐month landmark analysis: Kaplan–Meier estimates of OS and PFS in the HAIC (top) and TACE (bottom) subgroups. (B) 6‐month landmark analysis: Kaplan–Meier estimates of OS and PFS in the HAIC (top) and TACE (bottom) subgroups. (C) 12‐month landmark analysis: Kaplan–Meier estimates of OS and PFS in the HAIC (top) and TACE (bottom) subgroups. (D) 18‐month landmark analysis: Kaplan–Meier estimates of OS and PFS in the HAIC (top) and TACE (bottom) subgroups. (E) 24‐month landmark analysis: Kaplan–Meier estimates of OS and PFS in the HAIC (top) and TACE (bottom) subgroups.
**Table S1:** Baseline characteristics before and after PSM.
**Table S2:** Tumour response assessed by RECIST 1.1 and mRECIST.
**Table S3:** Cox model with Intervention_mode × PD‐1 timing interaction.
**Table S4:** Distribution of PD‐1 inhibitor types.
**Table S5:** Sensitivity analysis stratified by PD‐1 inhibitor type and ECOG PS.
**Table S6:** Treatment‐related adverse events stratified by locoregional therapy modality and PD‐1 inhibitor timing.

## Data Availability

The data that support the findings of this study are not publicly available due to their containing information that could compromise the privacy of research participants but are available from the corresponding author (Zhong‐wu Chen).
