# Supplementary material for: Impact of PD‐1 Inhibitor Timing Relative to Locoregional Therapy on Outcomes in uHCC Treated With Lenvatinib‐Based Triple Therapy
Source: Liver Int. 2026 Aug 27;46(10):e70847. doi: 10.1111/liv.70847 (PMC13522736; doi:10.1111/liv.70847)
Supplement: Supplementary file 1 [file LIV-46-0-s001.docx]

| **TableS1** Baseline characteristics before and after PSM | | | | | | | | | | | | | | | | | | |
| --- | --- | --- | --- | --- | --- | --- | --- | --- | --- | --- | --- | --- | --- | --- | --- | --- | --- | --- |
|  |  |  | | **HAIC group** | | | | | | |  |  | **TACE group** | | | | | |
| **Characteristic** |  | **Before PSM** | | | | |  | **After PSM** | | |  | **Before PSM** | | |  | **After PSM** | | |
|  | **Pre-LRT PD1**  N = 61 | | **Post-LRT PD1**  N = 124 | | **SMD** | **p-value** | **Pre-LRT PD1**  N = 53 | **Post-LRT PD1**  N = 53 | **SMD** | **p-value** | **Pre-LRT PD1**  N = 67 | **Post-LRT PD1**  N = 98 | **SMD** | **p-value** | **Pre-LRT PD1**  N = 55 | **Post-LRT PD1**  N = 55 | **SMD** | **p-value** |
| **Age, n (%)** |  | |  | | 0.080 | 0.08 |  |  | 0.080 | 0.08 |  |  | 0.555 | 0.001 |  |  | 0.040 | 1 |
| <60y | 37 (60.7%) | | 80 (64.5%) | |  |  | 37 (60.7%) | 80 (64.5%) |  |  | 49 (73.1%) | 46 (46.9%) |  |  | 38 (70.4%) | 37 (68.5%) |  |  |
| ≥60y | 24 (39.3%) | | 44 (35.5%) | |  |  | 24 (39.3%) | 44 (35.5%) |  |  | 18 (26.9%) | 52 (53.1%) |  |  | 16 (29.6%) | 17 (31.5%) |  |  |
| **Gender, n (%)** |  | |  | | 0.005 | 1 |  |  | 0.005 | 1 |  |  | 0.157 | 0.472 |  |  | 0.067 | 1 |
| Male | 56 (91.8%) | | 114 (91.9%) | |  |  | 56 (91.8%) | 114 (91.9%) |  |  | 60 (89.6%) | 92 (93.9%) |  |  | 50 (92.6%) | 49 (90.7%) |  |  |
| Female | 5 (8.2%) | | 10 (8.1%) | |  |  | 5 (8.2%) | 10 (8.1%) |  |  | 7 (10.4%) | 6 (6.1%) |  |  | 4 (7.4%) | 5 (9.3%) |  |  |
| **ECOG** |  | |  | | 0.254 | 0.143 |  |  | 0.043 | 1 |  |  | 0.036 | 1 |  |  | 0.061 | 1 |
| 0 | 42 (68.9%) | | 99 (79.8%) | |  |  | 39 (73.6%) | 40 (75.5%) |  |  | 58 (86.6%) | 86 (87.8%) |  |  | 48 (88.9%) | 49 (90.7%) |  |  |
| 1 | 19 (31.1%) | | 25 (20.2%) | |  |  | 14 (26.4%) | 13 (24.5%) |  |  | 9 (13.4%) | 12 (12.2%) |  |  | 6 (11.1%) | 5 (9.3%) |  |  |
| **Tbil (μmol/L), mean ± SD** | 20 ± 14 | | 16 ± 8 | | 0.343 | 0.017 | 18 ± 8 | 17 ± 8 | 0.114 | 0.559 | 16 ± 16 | 18 ± 24 | 0.072 | 0.663 | 16 ± 16 | 14 ± 7 | 0.013 | 0.945 |
| **HBV_infection, n (%)** |  | |  | | 0.165 | 0.371 |  |  | 0.043 | 1 |  |  | 0.070 | 0.84 |  |  | 0.054 | 1 |
| Negative | 16 (26.2%) | | 31 (25.0%) | |  |  | 13 (24.5%) | 14 (26.4%) |  |  | 8 (11.9%) | 14 (14.3%) |  |  | 7 (13.0%) | 8 (14.8%) |  |  |
| Positive | 45 (73.8%) | | 93 (75.0%) | |  |  | 40 (75.5%) | 39 (73.6%) |  |  | 59 (88.1%) | 84 (85.7%) |  |  | 47 (87.0%) | 46 (85.2%) |  |  |
| **HCV_infection, n (%)** |  | |  | | 0.175 | 0.527 |  |  | 0.196 | 1 |  |  | 0.141 | 0.738 |  |  | 0.000 | 1 |
| Negative | 59 (96.7%) | | 123 (99.2%) | |  |  | 52 (98.1%) | 53 (100.0%) |  |  | 65 (97.0%) | 97 (99.0%) |  |  | 53 (98.1%) | 53 (98.1%) |  |  |
| Positive | 2 (3.3%) | | 1 (0.8%) | |  |  | 1 (1.9%) | 0 (0.0%) |  |  | 2 (3.0%) | 1 (1.0%) |  |  | 1 (1.9%) | 1 (1.9%) |  |  |
| **HBV_DNA** **copies, n (%)** |  | |  | | 0.165 | 0.371 |  |  | 0.080 | 0.837 |  |  | 0.051 | 0.874 |  |  | 0.038 | 1 |
| ≤500 IU/mL | 25 (41.0%) | | 41 (33.1%) | |  |  | 19 (35.8%) | 17 (32.1%) |  |  | 25 (37.3%) | 39 (39.8%) |  |  | 21 (38.9%) | 20 (37.0%) |  |  |
| >500 IU/mL | 36 (59.0%) | | 83 (66.9%) | |  |  | 34 (64.2%) | 36 (67.9%) |  |  | 42 (62.7%) | 59 (60.2%) |  |  | 33 (61.1%) | 34 (63.0%) |  |  |
| **AFP, n (%)** |  | |  | | 0.051 | 0.866 |  |  | 0.116 | 0.69 |  |  | 0.117 | 0.562 |  |  | 0.000 | 1 |
| <400 ng/mL | 26 (42.6%) | | 56 (45.2%) | |  |  | 22 (41.5%) | 19 (35.8%) |  |  | 34 (50.7%) | 44 (44.9%) |  |  | 27 (50.0%) | 27 (50.0%) |  |  |
| ≥400 ng/mL | 35 (57.4%) | | 68 (54.8%) | |  |  | 31 (58.5%) | 34 (64.2%) |  |  | 33 (49.3%) | 54 (55.1%) |  |  | 27 (50.0%) | 27 (50.0%) |  |  |
| **Child-Pugh class, n (%)** |  | |  | | 0.217 | 0.223 |  |  | 0.047 | 1 |  |  | 0.277 | 0.144 |  |  | 0.118 | 0.759 |
| Child A | 47 (77.0%) | | 106 (85.5%) | |  |  | 43 (81.1%) | 42 (79.2%) |  |  | 61 (91.0%) | 80 (81.6%) |  |  | 49 (90.7%) | 47 (87.0%) |  |  |
| Child B | 14 (23.0%) | | 18 (14.5%) | |  |  | 10 (18.9%) | 11 (20.8%) |  |  | 6 (9.0%) | 18 (18.4%) |  |  | 5 (9.3%) | 7 (13.0%) |  |  |
| **Cirrhosis, n (%)** |  | |  | | 0.025 | 1 |  |  | 0.122 | 0.675 |  |  | 0.162 | 0.405 |  |  | 0.089 | 0.817 |
| No | 18 (29.5%) | | 38 (30.6%) | |  |  | 15 (28.3%) | 18 (34.0%) |  |  | 16 (23.9%) | 17 (17.3%) |  |  | 13 (24.1%) | 11 (20.4%) |  |  |
| Yes | 43 (70.5%) | | 86 (69.4%) | |  |  | 38 (71.7%) | 35 (66.0%) |  |  | 51 (76.1%) | 81 (82.7%) |  |  | 41 (75.9%) | 43 (79.6%) |  |  |
| **Tumorsize, n (%)** |  | |  | | 0.099 | 0.64 |  |  | 0.082 | 0.832 |  |  | 0.145 | 0.453 |  |  | 0.111 | 0.7 |
| <10 cm | 20 (32.8%) | | 35 (28.2%) | |  |  | 17 (32.1%) | 15 (28.3%) |  |  | 28 (41.8%) | 48 (49.0%) |  |  | 24 (44.4%) | 27 (50.0%) |  |  |
| ≥10 cm | 41 (67.2%) | | 89 (71.8%) | |  |  | 36 (67.9%) | 38 (71.7%) |  |  | 39 (58.2%) | 50 (51.0%) |  |  | 30 (55.6%) | 27 (50.0%) |  |  |
| **Tumornumber, n (%)** |  | |  | | 0.069 | 0.804 |  |  | 0.047 | 1 |  |  | 0.241 | 0.177 |  |  | 0.040 | 1 |
| Single | 13 (21.3%) | | 23 (18.5%) | |  |  | 11 (20.8%) | 10 (18.9%) |  |  | 26 (38.8%) | 27 (27.6%) |  |  | 17 (31.5%) | 18 (33.3%) |  |  |
| Multiple | 48 (78.7%) | | 101 (81.5%) | |  |  | 42 (79.2%) | 43 (81.1%) |  |  | 41 (61.2%) | 71 (72.4%) |  |  | 37 (68.5%) | 36 (66.7%) |  |  |
| **PVTT, n (%)** |  | |  | | 0.041 | 0.995 |  |  | 0.047 | 0.996 |  |  | 0.338 | 0.194 |  |  | 0.025 | 0.716 |
| VP0 | 12 (19.7%) | | 23 (18.5%) | |  |  | 11 (20.8%) | 11 (20.8%) |  |  | 32 (47.8%) | 59 (60.2%) |  |  | 27 (50.0%) | 30 (55.6%) |  |  |
| VP2 | 9 (14.8%) | | 18 (14.5%) | |  |  | 7 (13.2%) | 7 (13.2%) |  |  | 10 (14.9%) | 13 (13.3%) |  |  | 9 (16.7%) | 10 (18.5%) |  |  |
| VP3 | 22 (36.1%) | | 47 (37.9%) | |  |  | 20 (37.7%) | 21 (39.6%) |  |  | 17 (25.4%) | 22 (22.4%) |  |  | 15 (27.8%) | 10 (18.5%) |  |  |
| VP4 | 18 (29.5%) | | 36 (29.0%) | |  |  | 15 (28.3%) | 14 (26.4%) |  |  | 8 (11.9%) | 4 (4.1%) |  |  | 3 (5.6%) | 4 (7.4%) |  |  |
| **EHS, n (%)** |  | |  | | 0.218 | 0.229 |  |  | 0.081 | 0.835 |  |  | 0.086 | 0.726 |  |  | 0.185 | 0.474 |
| No | 45 (73.8%) | | 79 (63.7%) | |  |  | 37 (69.8%) | 35 (66.0%) |  |  | 53 (79.1%) | 74 (75.5%) |  |  | 41 (75.9%) | 45 (83.3%) |  |  |
| Yes | 16 (26.2%) | | 45 (36.3%) | |  |  | 16 (30.2%) | 18 (34.0%) |  |  | 14 (20.9%) | 24 (24.5%) |  |  | 13 (24.1%) | 9 (16.7%) |  |  |
| **PD_1type** |  | |  | | 0.512 | 0.004 |  |  | 0.045 | 0.973 |  |  | 0.368 | 0.076 |  |  | 0.103 | 0.866 |
| Camrelizumab | 22 (36.1%) | | 69 (55.6%) | |  |  | 19 (35.8%) | 20 (37.7%) |  |  | 35 (52.2%) | 37 (37.8%) |  |  | 24 (44.4%) | 24 (44.4%) |  |  |
| Tislelizumab | 18 (29.5%) | | 37 (29.8%) | |  |  | 18 (34.0%) | 17 (32.1%) |  |  | 21 (31.3%) | 48 (49.0%) |  |  | 20 (37.0%) | 18 (33.3%) |  |  |
| Sintilimab | 21 (34.4%) | | 18 (14.5%) | |  |  | 16 (30.2%) | 16 (30.2%) |  |  | 11 (16.4%) | 13 (13.3%) |  |  | 10 (18.5%) | 12 (22.2%) |  |  |
| **LRT_number** |  | |  | | 0.182 | 0.315 |  |  | 0.078 | 0.841 |  |  | 0.378 | 0.029 |  |  | 0.149 | 0.563 |
| <3 | 25 (41.0%) | | 40 (32.3%) | |  |  | 21 (39.6%) | 19 (35.8%) |  |  | 37 (55.2%) | 36 (36.7%) |  |  | 27 (50.0%) | 23 (42.6%) |  |  |
| ≥3 | 36 (59.0%) | | 84 (67.7%) | |  |  | 32 (60.4%) | 34 (64.2%) |  |  | 30 (44.8%) | 62 (63.3%) |  |  | 27 (50.0%) | 31 (57.4%) |  |  |

Abbreviation: HAIC hepatic arterial infusion chemotherapy; TACE transarterial chemoembolization; PSM propensity score matching; SMD: Standardized Mean Difference; AFP Alpha-Fetoprotein; PVTT Portal Vein Tumor Thrombus; EHS Extrahepatic Spread; LRT locoregional therapy.

| Table S2. Tumor response assessed by RECIST 1.1 and mRECIST | | | | | | | | | | |
| --- | --- | --- | --- | --- | --- | --- | --- | --- | --- | --- |
|  | **RECIST 1.1** | | | | | **mRECIST** | | | | |
| **Characteristic** | **HAIC + Pre-LRT PD1**, N = 61 | **HAIC + Post-LRT PD1**, N = 124 | **TACE + Pre-LRT PD1**, N = 67 | **TACE + Post-LRT PD1**, N = 98 | **p-value**^1^ | **HAIC + Pre-LRT PD1**, N = 61 | **HAIC + Post-LRT PD1**, N = 124 | **TACE + Pre-LRT PD1**, N = 67 | **TACE + Post-LRT PD1**, N = 98 | **p-value**^1^ |
| **Best response** |  |  |  |  | 0.002 |  |  |  |  | 0.6 |
| CR | 4 (6.6%) | 2 (1.6%) | 1 (1.5%) | 2 (2.0%) |  | 11 (18.0%) | 23 (18.5%) | 4 (6.0%) | 6 (6.1%) |  |
| PR | 32 (52.5%) | 60 (48.4%) | 27 (40.3%) | 28 (28.6%) |  | 31 (50.8%) | 60 (48.4%) | 43 (64.2%) | 64 (65.3%) |  |
| SD | 21 (34.4%) | 50 (40.3%) | 33 (49.3%) | 53 (54.1%) |  | 15 (24.6%) | 28 (22.6%) | 14 (20.9%) | 12 (12.2%) |  |
| PD | 4 (6.6%) | 12 (9.7%) | 6 (9.0%) | 15 (15.3%) |  | 4 (6.6%) | 13 (10.5%) | 6 (9.0%) | 16 (16.3%) |  |
| **ORR, n (%)** | 36 (59.0%) | 62 (50.0%) | 28 (41.8%) | 30 (30.6%) | 0.002 | 42 (68.9%) | 83 (66.9%) | 47 (70.1%) | 70 (71.4%) | >0.9 |
| **DCR, n (%)** | 57 (93.4%) | 112 (90.3%) | 61 (91.0%) | 83 (84.7%) | 0.3 | 57 (93.4%) | 111 (89.5%) | 61 (91.0%) | 82 (83.7%) | 0.2 |
| ^1^For ordered best tumor response (CR/PR/SD/PD), Kruskal-Wallis H test was used for between-group comparison. For binary endpoints (ORR, DCR), Pearson’s chi-squared test was applied for four-group comparison. | | | | | | | | | | |
| **Table S3**. Cox model with Intervention_mode × PD-1 timing interaction | | | | | | | | | | |

| **Characteristic** | **HR** | **95% CI** | **p-value** |
| --- | --- | --- | --- |
| **OS** |  |  |  |
| Intervention_mode |  |  |  |
| HAIC | — | — |  |
| TACE | 1.87 | 0.92, 3.82 | 0.084 |
| PD_1 timing |  |  |  |
| Pre-LRT PD1 therapy | — | — |  |
| Post-LRT PD1 therapy | 2.22 | 1.19, 4.14 | 0.013 |
| Intervention_mode * PD_1 timing |  |  |  |
| TACE * Post-LRT PD1 therapy | 0.41 | 0.18, 0.93 | 0.033 |
| Interaction P = 0.030 |  |  |  |
| **PFS** |  |  |  |
| Intervention_mode |  |  |  |
| HAIC | — | — |  |
| TACE | 1.25 | 0.80, 1.95 | 0.3 |
| PD_1 timing |  |  |  |
| Pre-LRT PD1 therapy | — | — |  |
| Post-LRT PD1 therapy | 1.40 | 0.94, 2.07 | 0.10 |
| Intervention_mode * PD_1 timing |  |  |  |
| TACE * Post-LRT PD1 therapy | 0.68 | 0.40, 1.18 | 0.2 |
| Interaction P (LRT) = 0.167; P-values were calculated using Wald's tests for individual regression coefficients. Interaction P-values were obtained from likelihood ratio tests comparing Cox models with and without the interaction term between intervention modality and PD-1 timing. Multivariable Cox regression models were adjusted for prespecified clinical covariates, including age, sex, ECOG performance status, HBV infection status, HCV infection status, HBV-DNA level, AFP level, Child-Pugh class, cirrhosis status, total bilirubin, tumor size, tumor number, PVTT grade, EHS, PD-1 inhibitor type, and the number of LRT. | | | |

| Table S4. Distribution of PD-1 inhibitor types | | | | | | | | |
| --- | --- | --- | --- | --- | --- | --- | --- | --- |
|  | **HAIC** | | | | **TACE** | | | |
| **Characteristic** | **Overall**, N = 185 | **Pre-LRT PD1 therapy**, N = 61 | **Post-LRT PD1 therapy**, N = 124 | **p-value**^1^ | **Overall**, N = 165 | **Pre-LRT PD1 therapy**, N = 67 | **Post-LRT PD1 therapy**, N = 98 | **p-value**^1^ |
| PD_1type |  |  |  | 0.004 |  |  |  | 0.076 |
| Camrelizumab | 91 (49.2%) | 22 (36.1%) | 69 (55.6%) |  | 72 (43.6%) | 35 (52.2%) | 37 (37.8%) |  |
| Tislelizumab | 55 (29.7%) | 18 (29.5%) | 37 (29.8%) |  | 69 (41.8%) | 21 (31.3%) | 48 (49.0%) |  |
| Sintilimab | 39 (21.1%) | 21 (34.4%) | 18 (14.5%) |  | 24 (14.5%) | 11 (16.4%) | 13 (13.3%) |  |
| ^1^ Pearson's Chi-squared test | | | | | | | | |

| **Table S5.** Sensitivity analysis stratified by PD-1 inhibitor type and ECOG PS | | | | | | |  |
| --- | --- | --- | --- | --- | --- | --- | --- |
| **Characteristic** | **Endpoint** | **PD_1 type** | **N** | **HR** | **95% CI** | **p-value^1^** | |
| HAIC | OS | Camrelizumab | 91 | 1.14 | 0.5, 2.59 | 0.754 |  |
| HAIC | OS | Tislelizumab | 55 | 3.61 | 1.23, 10.57 | 0.019 |  |
| HAIC | OS | Sintilimab | 39 | 3.42 | 0.69, 16.99 | 0.133 |  |
| HAIC | PFS | Camrelizumab | 91 | 0.97 | 0.54, 1.72 | 0.906 |  |
| HAIC | PFS | Tislelizumab | 55 | 2.52 | 1.22, 5.17 | 0.012 |  |
| HAIC | PFS | Sintilimab | 39 | 1.43 | 0.68, 3.02 | 0.345 |  |
| TACE | OS | Camrelizumab | 72 | 0.69 | 0.34, 1.41 | 0.310 |  |
| TACE | OS | Tislelizumab | 69 | 3.31 | 0.99, 11.14 | 0.052 |  |
| TACE | PFS | Camrelizumab | 72 | 0.68 | 0.39, 1.18 | 0.167 |  |
| TACE | PFS | Tislelizumab | 69 | 1.45 | 0.76, 2.78 | 0.263 |  |
| HAIC | OS | ECOG PS =0 | 141 | 2.49 | 1.17, 5.3 | 0.018 |  |
| HAIC | OS | ECOG PS =1 | 44 | 2.31 | 0.84, 6.31 | 0.103 |  |
| HAIC | PFS | ECOG PS =0 | 141 | 1.52 | 0.97, 2.38 | 0.065 |  |
| HAIC | PFS | ECOG PS =1 | 44 | 1.11 | 0.55, 2.22 | 0.770 |  |
| TACE | OS | ECOG PS =0 | 144 | 1.01 | 0.58, 1.75 | 0.975 |  |
| TACE | OS | ECOG PS =1 | 21 | 1.17 | 0.33, 4.18 | 0.810 |  |
| TACE | PFS | ECOG PS =0 | 144 | 1 | 0.67, 1.5 | 0.989 |  |
| TACE | PFS | ECOG PS =1 | 21 | 0.86 | 0.32, 2.31 | 0.764 |  |
| **^1^** Wald's test | | | | | | |  |

| **Table S6.** Treatment-Related Adverse Events Stratified by Locoregional Therapy Modality and PD-1 Inhibitor Timing | | | | | | | | | | | | |
| --- | --- | --- | --- | --- | --- | --- | --- | --- | --- | --- | --- | --- |
|  | **Any grade** | | | | | | **Grade 3/4** | | | | | |
| **Characteristic** | **HAIC +** **Len+Pre-LRT PD1**, N = 61 | **HAIC + Len+Post-LRT PD1**, N = 124 | **P value^1^** | **TACE +Len+ Pre-LRT PD1**, N = 67 | **TACE + Len+Post-LRT PD1**, N = 98 | **P value^1^** | **HAIC + Len+Pre-LRT PD1**, N = 61 | **HAIC + Len+Post-LRT PD1**, N = 124 | **P value^1^** | **TACE +Len+ Pre-LRT PD1**, N = 67 | **TACE + Len+Post-LRT PD1**, N = 98 | **P value^1^** |
| Abdominal pain | 14 (23.0%) | 32 (25.8%) | 0.721 | 44 (65.7%) | 71 (72.4%) | 0.391 | 2 (3.3%) | 2 (1.6%) | 0.600 | 10 (14.9%) | 18 (18.4%) | 0.391 |
| Fever | 9 (14.8%) | 28 (22.6%) | 0.245 | 31 (46.3%) | 61 (62.2%) | 0.055 | 0 (0%) | 0 (0%) | NA | 1 (1.5%) | 2 (2.0%) | 0.055 |
| Nausea and vomiting | 42 (68.9%) | 72 (58.1%) | 0.198 | 22 (32.8%) | 36 (36.7%) | 0.623 | 4 (6.6%) | 8 (6.5%) | >0.9 | 2 (3.0%) | 3 (3.1%) | 0.623 |
| Diarrhoea | 16 (26.2%) | 36 (29.0%) | 0.731 | 20 (29.9%) | 25 (25.5%) | 0.595 | 1 (1.6%) | 3 (2.4%) | >0.9 | 1 (1.5%) | 2 (2.0%) | 0.595 |
| Decreased appetite | 22 (36.1%) | 56 (45.2%) | 0.270 | 26 (38.8%) | 35 (35.7%) | 0.744 | 1 (1.6%) | 3 (2.4%) | >0.9 | 1 (1.5%) | 1 (1.0%) | 0.744 |
| Elevated AST | 34 (55.7%) | 68 (54.8%) | >0.9 | 54 (80.6%) | 70 (71.4%) | 0.203 | 6 (9.8%) | 9 (7.3%) | 0.573 | 12 (17.9%) | 13 (13.3%) | 0.203 |
| Elevated ALT | 30 (49.2%) | 59 (47.6%) | 0.876 | 48 (71.6%) | 62 (63.3%) | 0.314 | 5 (8.2%) | 6 (4.8%) | 0.509 | 9 (13.4%) | 9 (9.2%) | 0.314 |
| Hyperbilirubinaemia | 18 (29.5%) | 28 (22.6%) | 0.366 | 24 (35.8%) | 29 (29.6%) | 0.403 | 2 (3.3%) | 2 (1.6%) | 0.600 | 3 (4.5%) | 3 (3.1%) | 0.403 |
| Hypoalbuminaemia | 16 (26.2%) | 39 (31.5%) | 0.498 | 18 (26.9%) | 23 (23.5%) | 0.714 | 1 (1.6%) | 2 (1.6%) | >0.9 | 1 (1.5%) | 1 (1.0%) | 0.714 |
| Neutropenia | 10 (16.4%) | 73 (58.9%) | <0.001 | 11 (16.4%) | 17 (17.3%) | >0.9 | 1 (1.6%) | 21 (16.9%) | 0.001 | 1 (1.5%) | 3 (3.1%) | >0.9 |
| Thrombocytopenia | 26 (42.6%) | 48 (38.7%) | 0.635 | 32 (47.8%) | 42 (42.9%) | 0.633 | 4 (6.6%) | 9 (7.3%) | >0.9 | 5 (7.5%) | 6 (6.1%) | 0.633 |
| Anaemia | 14 (23.0%) | 40 (32.3%) | 0.230 | 16 (23.9%) | 19 (19.4%) | 0.562 | 1 (1.6%) | 5 (4.0%) | 0.665 | 1 (1.5%) | 1 (1.0%) | 0.562 |
| Hypertension | 24 (39.3%) | 52 (41.9%) | 0.753 | 28 (41.8%) | 41 (41.8%) | >0.9 | 5 (8.2%) | 10 (8.1%) | >0.9 | 6 (9.0%) | 8 (8.2%) | >0.9 |
| Fatigue | 20 (32.8%) | 54 (43.5%) | 0.202 | 24 (35.8%) | 31 (31.6%) | 0.616 | 1 (1.6%) | 4 (3.2%) | >0.9 | 1 (1.5%) | 2 (2.0%) | 0.616 |
| Proteinuria | 14 (23.0%) | 28 (22.6%) | >0.9 | 18 (26.9%) | 20 (20.4%) | 0.352 | 2 (3.3%) | 2 (1.6%) | 0.600 | 2 (3.0%) | 2 (2.0%) | 0.352 |
| Hypothyroidism | 12 (19.7%) | 21 (16.9%) | 0.685 | 13 (19.4%) | 15 (15.3%) | 0.531 | 0 (0%) | 0 (0%) | NA | 0 (0%) | 0 (0%) | 0.531 |
| Rash | 9 (14.8%) | 17 (13.7%) | 0.826 | 10 (14.9%) | 11 (11.2%) | 0.487 | 1 (1.6%) | 0 (0.0%) | 0.330 | 1 (1.5%) | 1 (1.0%) | 0.487 |
| Hand–foot skin reaction | 18 (29.5%) | 33 (26.6%) | 0.728 | 22 (32.8%) | 24 (24.5%) | 0.289 | 2 (3.3%) | 2 (1.6%) | 0.600 | 3 (4.5%) | 2 (2.0%) | 0.289 |
| Immune-related hepatitis | 3 (4.9%) | 11 (8.9%) | 0.395 | 3 (4.5%) | 3 (3.1%) | 0.688 | 1 (1.6%) | 3 (2.4%) | >0.9 | 1 (1.5%) | 1 (1.0%) | 0.688 |
| ^1^*p* values were calculated using a two-sided *χ*2 test | | | | | | | | | | | | |

**Fig S1.** Subgroup analyses of PFS (A) and OS (B) according to PD-1 inhibitor timing in all patients. Abbreviation: HAIC hepatic arterial infusion chemotherapy; TACE transarterial chemoembolization; PSM propensity score matching; AFP Alpha-Fetoprotein; PVTT Portal Vein Tumor Thrombus; EHS Extrahepatic Spread.

**Fig S2.** Landmark survival analysis in the HAIC and TACE subgroups. (A) 3-month landmark analysis: Kaplan–Meier estimates of OS and PFS in the HAIC (top) and TACE (bottom) subgroups. (B) 6-month landmark analysis: Kaplan–Meier estimates of OS and PFS in the HAIC (top) and TACE (bottom) subgroups. (C) 12-month landmark analysis: Kaplan–Meier estimates of OS and PFS in the HAIC (top) and TACE (bottom) subgroups. (D) 18-month landmark analysis: Kaplan–Meier estimates of OS and PFS in the HAIC (top) and TACE (bottom) subgroups. (E) 24-month landmark analysis: Kaplan–Meier estimates of OS and PFS in the HAIC (top) and TACE (bottom) subgroups.
